# Ubiquitin ligase Cbl-b represses IGF-I-induced epithelial mesenchymal transition via ZEB2 and microRNA-200c regulation in gastric cancer cells

**DOI:** 10.1186/1476-4598-13-136

**Published:** 2014-06-02

**Authors:** Heming Li, Ling Xu, Ce Li, Lei Zhao, Yanju Ma, Huachuan Zheng, Zhi Li, Ye Zhang, Ruoyu Wang, Yunpeng Liu, Xiujuan Qu

**Affiliations:** 1 Department of Medical Oncology, the First Hospital of China Medical University, NO.155, North Nanjing Street, Heping District, Shenyang 110001, China; 2 Cancer Research Center, the First Affiliated Hospital of Liaoning Medical University, Jinzhou 121001, China; 3 Department of Medical Oncology, the Affiliated Zhongshan Hospital of Dalian University, Dalian 116001, China

**Keywords:** IGF-I, EMT, ZEB2, Cbl-b, microRNA-200c

## Abstract

**Background:**

Insulin-like growth factor I (IGF-I) can induce epithelial mesenchymal transition (EMT) in many epithelial tumors; however, the molecular mechanism by which this occurs is not clearly understood. Additionally, little is known about the involvement of IGF-I in gastric cancer.

**Methods:**

Two gastric cancer cell lines were treated with IGF-I to induce EMT and levels of transcription factor ZEB2 and microRNA-200c (miR-200c) were measured. Cells were treated with Akt/ERK inhibitors to investigate the role of these pathways in IGF-I-mediated EMT. Transfection of shRNA plasmids was used to silence the ubiquitin ligase Cbl-b to assess its involvement in this process. The relationship between IGF-IR and Cbl-b expression, and the effect of IGF-IR and Cbl-b on metastasis were analyzed in primary gastric adenocarcinoma patients.

**Results:**

IGF-I-induced gastric cancer cell EMT was accompanied by ZEB2 up-regulation. Furthermore, both Akt/ERK inhibitors and knockdown of Akt/ERK gene reversed IGF-I-induced ZEB2 up-regulation and EMT through up-regulation of miR-200c, suggesting the involvement of an Akt/ERK-miR-200c-ZEB2 axis in IGF-I-induced EMT. The ubiquitin ligase Cbl-b also ubiquitinated and degraded IGF-IR and inhibited the Akt/ERK-miR-200c-ZEB2 axis, leading to the repression of IGF-I-induced EMT. There was a significant negative correlation between the expression of IGF-IR and Cbl-b in gastric cancer patient tissues (r = -0.265, p < 0.05). More of patients with IGF-IR-positive expression and Cbl-b-negative expression were with lymph node metastasis (p < 0.001).

**Conclusions:**

Together, these findings demonstrate that the ubiquitin ligase Cbl-b represses IGF-I-induced EMT, likely through targeting IGF-IR for degradation and further inhibiting the Akt/ERK-miR-200c-ZEB2 axis in gastric cancer cells.

## Introduction

Gastric cancer is one of the most common causes of cancer death worldwide [1]. Additionally, most patients are diagnosed with advanced metastatic disease; the 5-year survival rate is approximately 10–15% [2]. Although chemotherapy, radiotherapy, and targeted therapy have improved the response rate, patients with metastatic gastric cancer remain have a poor prognosis [2,3]. Contributing to this problem is the lack of effective biomarkers for metastasis prediction. Therefore, it is necessary and urgent to explore the mechanisms of metastasis in gastric cancer.

Tumor metastasis is a multi-step dynamic process involving multiple factors and genes. Recent evidence indicates that epithelial-to-mesenchymal transition (EMT) is a key driver of progression and metastasis in tumors, including gastric cancer, breast cancer, hepatocellular carcinoma, and prostate cancer [4-7]. In this process, epithelial cells lose cell-cell adhesions and acquire properties of mesenchymal cells, namely enhanced migratory and invasive abilities [8]. Many growth factors are involved in the initiation of EMT, including the insulin-like growth factor-I receptor (IGF-IR)/ligand system that has been reported to increase the metastatic potential of prostate and breast cancer cells [5,6]. Consistently, clinical studies have observed increased baseline IGF-I serum levels in patients with gastric cancer and overexpression of IGF-IR is a significant predictive value for poor survival in such patients [9,10]. However, whether IGF-I promotes gastric cancer metastasis by EMT, and the mechanisms by which this may occur remain unclear.

Ubiquitination is a post-translational modification that targets cellular proteins for degradation [11]. Almost all cellular processes are regulated by the ubiquitin proteasome system, including EMT [12]. Cbl-b is the second member of the E3 ubiquitin ligase Cbl family, and our group and others have revealed that Cbl-b regulates cancer cell proliferation, drug sensitivity, and migration [13-15]. Knock-down of Cbl-b enhances epidermal growth factor-induced disruption of human mammary epithelial cell adherens junctions (AJs) and cell motility [16]. The inducible up-regulation of c-Cbl and Cbl-b affects cell adhesion through regulation of the adhesion-related kinases Pyk2 and Paxillin in HL-60 cell differentiation [17]. Moreover, Cbl-b can also degrade the IGF-I signaling intermediate IRS-1 and reduce protein synthesis in unloading-induced muscle atrophy [18]. Our recent published data demonstrated that Cbl-b suppressed TRAIL-induced IGF-IR activation by regulating its distribution in the lipid raft [19]. However, whether Cbl-b can target IGF-IR for degradation and if this process is involved in IGF-I-induced EMT require further investigations.

Here, we reveal the existence of an Akt/ERK-miR-200c-ZEB2 axis in IGF-I-induced EMT in gastric cancer cells. Furthermore, the ubiquitin ligase Cbl-b ubiquitinated IGF-IR and repressed IGF-I-induced EMT through negative regulation of this Akt/ERK-miR-200c-ZEB2 axis.

## Materials and methods

### Cell cultures

Human gastric cell lines MGC803, SGC-7901 were obtained from the Type Culture Collection of the Chinese Academy of Sciences (China). The cells were maintained in RPMI-1640 medium (Gibco) with 10% heat-inactivated fetal bovine serum (FBS), penicillin (100 U/mL) and streptomycin (100 mg/mL) in an atmosphere of 95% air and 5% CO_2_ at 37°C. The cells were sub-cultured every 2–3 days and harvested in their logarithmic phase of growth.

### Reagents and antibodies

Recombinant human IGF-I was purchased from R&D System (Wiesbaden, Germany). The dual IGF-IR/IR inhibitor OSI-906 was purchased from SelleckBio (USA). Specific PI3K/Akt inhibitor LY294002 was purchased from Sigma (St. Louis, MO), and specific ERK1/2 inhibitor PD98059 was purchased from Promega (Madison, WI). Proteasome inhibitor bortezomib (PS-341) was purchased from Millenium Pharmaceuticals Inc (Cambridge, MA, USA). Anti-E-cadherin, anti-Vimentin, anti-ZEB1, anti-IGF-IR, anti-phospho-IGF-IR (Tyr1131), anti-phospho-GSK-3β, anti-GSK-3β and anti-phospho-P53 (Ser15) were purchased from Cell Signaling Technology (Beverly, MA). Anti-Snail and anti-Twist2 were purchased from Abcam (Cambridge, MA). All the other antibodies were purchased from Santa Cruz Biotechnology (USA).

### Patients and tissue samples

A total of 50 lymph node metastasis and 50 non-lymph node metastasis surgically resected primary gastric adenocarcinoma patient specimens were obtained from the First Hospital of China Medical University between Jan 1st 2007 and Dec 31st 2008. Age, sex, pTNM stage and Lauren grade were evaluated following medical charts and pathology records. pTNM stage was examined according to the 7th edition of AJCC cancer staging manual. Lauren grade was reference to WHO classification. No patients had received any neoadjuvant chemotherapy and radiotherapy. All research involving human participants were approved by the Ethics Committee of China Medical University. Written informed consents were obtained from all the participants in accordance with the Helsinki Declaration.

### Immunohistochemistry

One hundred of formalin-fixed, paraffin-embedded primary gastric cancer tissues were cut into 3-mm sections. All sections were de-paraffinized in xylene and dehydrated through a graduated alcohol series followed by the standard procedure for the S-P immunohistochemical kit (Fuzhou Maixin Biological Technology Ltd., Fujian, China). Sections were incubated with anti-Cbl-b or anti-IGF-IR in PBS at 4°C overnight in a moist box. 3, 30-diamino-benzidine tetrahydrochloride (DAB kit; Fuzhou Maixin Biological Technology Ltd., Fujian, China) was used for immune complex visualization. The staining was evaluated by scanning the entire tissue specimen under low magnification (×10) and confirmed under high magnification (×20 and × 40). The protein expression was visualized and classified based on the percentage of positive cells and the intensity of staining. Tumors with less than 10% Cbl-b or IGF-IR expression were regarded as negative. Immunostaining was considered as positive when more than 10% of the neoplastic cells were stained. Final scores were assigned by two independent pathologists.

### Western blot and immunoprecipitation assay

Western blot analysis was performed as described in our previous studies [15]. Briefly, samples were solubilized in 1% Triton lysis buffer on ice or in RIPA buffer. For immunoprecipitation, the collected cell lysates were incubated with the indicated antibodies (1–4 μg) or immunoglobulin-G (Cell Signaling Technology, Beverly, MA) and precleared protein G-agarose beads overnight at 4°C. On the other day, the immunoprecipitates were washed extensively with lysis buffer for four times. For the preparation of total cell lysates, the monolayers were lysed directly as described in western blot analysis. Both immunoprecipitated proteins and cell lysates were then eluted by boiling water at 100°C for 5 min with 3 × sampling buffer. Total proteins were subjected to SDS-polyacrylamide gel electrophoresis and electronically transferred to nitrocellulose membranes. After blocking with 5% skim milk in TBST buffer, the blots were incubated in the primary antibodies followed by secondary antibodies as indicated time. Proteins were detected using an enhanced chemiluminescence reagent (SuperSignal Western Pico Chemiluminescent Substrate; Pierce, USA). The final result was analyzed by NIH Image J software.

### Reverse-transcription-polymerase chain reaction (RT-PCR)

The cells were cultured and harvested at the indicated times. Total RNA was isolated with the RNeasy mini kit (Qiagen, Carlsbad, CA, USA). RT-PCR was performed with primer pairs for ZEB2: forward (5′-CGCTTGACATCACTGAAGGA-3′) and reverse (5′-CTTGCCACACTC TGTGCATT-3′). For actin as control: forward (5′-GTGGGG CGCCCCAGGCACCA-3′) and reverse (5′-CTCCTTAATGTCACGCACGATTTC- 3′). PCR conditions were 95°C for 5 min; 31 cycles of 95°C for 30 s, 55°C for 45 s, 72°C for 40 s; one cycle of 72°C for 10 min. Then the amplified products were separated on 1.5% agarose gels, and stained with ethidium bromide and visualized under UV illumination.

### Quantitative reverse transcription real-time PCR (qRT-PCR)

Total RNA was extracted as mentioned above. For microRNAs, The One Step PrimeScript® miRNA cDNA Synthesis Kit (Takara, Japan) was used for RNA reverse transcription. Relative expression of microRNA was calculated via the comparative cycle threshold (Ct) method, and the expression of U6 small nuclear RNA was used as reference. The sequence-specific forward primers for mature miR-200c was: 5′-ACACTCCAGCTGGGTAATACTG CCGGGTAA-3′ and for U6 internal control was: forward (5′-GCTTCGGCAGCACATAT ACTAAAAT-3′) and reverse (5′-CGCTTCACGAATTTGCGTGTCAT-3′), respectively. The Uni-miR qPCR Primer was included in the kit. SYBR® Premix Ex Taq™ II (Perfect Real Time) (Takara, Japan) was used for monitoring the amount of miRNA. The PCR conditions were 30 s at 95°C, followed by 45 cycles at 95°C for 5 s and 58°C for 25 s. The threshold cycle and 2^-ΔΔCt^ method were used for calculating the relative amount of the target RNA.

### MicroRNA microarray

The expression levels of 847 human and 609 mouse microRNAs were quantitated using a GeneChip miRNA Array (Affymetrix, Santa Clara, CA) according to the manufacturer’s instructions by Gene Tech Biotechnology Company (Shanghai, China). In brief, total RNA (1 μg) was extracted with miRNeasy Mini Kit (Qiagen) and labeled with a FlashTag Biotin RNA Labeling kit (Genisphere, Hatfield, PA). Then the labeled RNA was injected onto the microarrays and incubated at 48°C for 16 hours. After washing and staining, the signals were obtained using a GeneChip Scanner 3000 (Affymetrix, Santa Clara, CA). Data were normalized using the RMA algorithm.

### Migration assay

Migration assay was performed using Boyden chambers and polycarbonate inserts with 8-um pore size membranes. The cells (1 × 10^4^ cells/well) were seeded into the upper chamber with 200 μL serum-free RPMI 1640 medium with or without IGF-I (100 ng/mL). Then the upper chamber was inserted into the lower chamber of 24-well culture dishes with 500 μL of RPMI 1640 containing 2.5% FBS. After incubation for 48 hours, the culture media in the upper chamber and non-migrated cells on the inner side of the membrane were carefully removed with a cotton swab. After dried for 1 hour at room temperature, the migrated cells onto the outer side of the membrane were fixed with 4% formaldehyde for 1 min and stained with 0.1% Giemsa stain solution for 2 hours. Then the migrated cells were counted in five different fields at × 10 magnification under the microscope.

### Immunofluorescence

The cells were seeded in Lab-Tek chamber slides (Nunc S/A, Polylabo, Strasbourg, France). After starved overnight, the cells were treated with or without IGF-I (100 ng/mL) for 48 hours and fixed in 3.3% paraformaldehyde for 15 min, permeabilized with 0.2% Triton X-100 for 5 min, blocked with 5% bovine serum albumin (BSA) for 1 hour and then incubated with anti-E-cadherin and anti-Vimentin antibody overnight at 4°C. The next day, Alexa Fluor 546-conjugated goat anti-rabbit IgG or Alexa Fluor 488-conjugated goat anti-rabbit IgG (Molecular Probes) were added in blocking solution for 1 hour at room temperature in the dark. 4′6′-diamidino-2- phenylindole was used to stain nuclei for 5 min. After mounted with the Slow Fade Antifade Kit (Molecular Probes, Eugene, OR, USA), the cells were visualized by fluorescence microscopy (BX61, Olympus, Japan).

### Small interfering RNA transfections

Cells were seeded at a density of 3 × 10^5^ cells/well in 6-well plates. The cells were transfected with siRNAs using Lipofectamine 2000 (Invitrogen, Carlsbad, CA, USA) following the manufacturer’s instructions. Two siRNA sequences (Qiagen Inc., Valencia, CA) each for ERK1 and ERK2 were as follows: ERK1: 5′-CGUCUAAUAUAUAAAUAUA dTdT-3′ (sense), 5′-UAUAUUUAUAUAUUAGACGdGdG-3′ (antisense); ERK2: 5′-CACU UGUCAAGAAGCGUUAdTdT-3′ (sense), 55′-UAACGCUUCUUGACAAGUGdTdT-3′ (anti-sense). The Akt siRNA was obtained from Sigma (MO, USA) and siRNA sequence was: 5′-GAGACUGACACCAGGUAUUdT dT-3′ (sense), 5′-AAUACCUGGUGUCAGUCUCdT dT-3′ (anti-sense). The control sequence was: AATTCTCCGAACGTGTCACGT. Western blot analysis was used to verify gene-silencing efficiency.

### Plasmid construction and stable cell lines establishment

The method of plasmid construction is performed as described previously [20].

### Statistical analysis

All the presented data were expressed as the mean ± SD and representative results were from at least three independent experiments. Statistical comparisons were calculated by Student’s two-tailed *t*-test. The correlation between Cbl-b and IGF-IR expression was assessed using Spearman rank correlation for continuous variables. The effect of Cbl-b and IGF-IR on metastasis was analyzed by Fisher exact test for tables. p < 0.05 was considered statistically significant. Statistical analysis was carried out using SPSS 18.0 software package (SPSS, Inc., Chicago, IL, USA).

## Results

### IGF-I-induced EMT in gastric cancer cells

**Figure 1:**
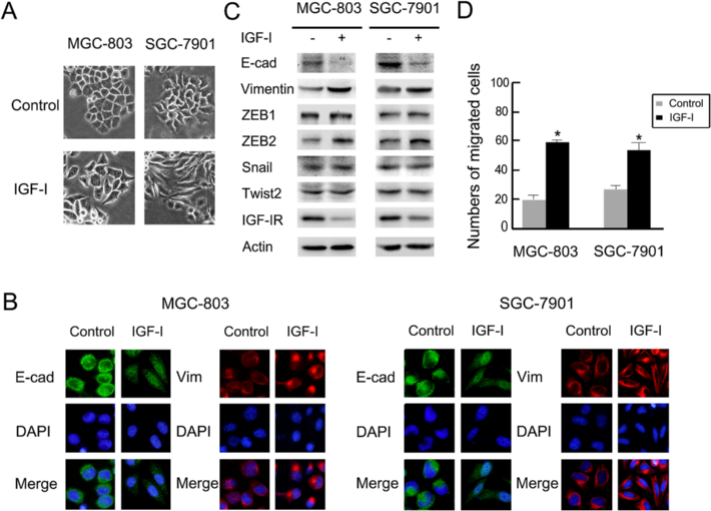
**IGF-I induced EMT and enhanced the migration ability in gastric cancer cells.** MGC-803 and SGC-7901 cells were serum-starved overnight and then treated with or without 100 ng/mL of IGF-I for 48 h. **(A)** Photos were taken at × 20 magnification. **(B)** The cells were stained with antibodies to E-cadherin (green), Vimentin (red), and nuclei was stained with 4′,6′-diamidino-2-phenylindole (DAPI). Images were captured by fluorescence microscopy at × 40 magnification. **(C)** Cell lysates were collected for Western blot analysis. **(D)** The migration assays were performed using the Boyden chamber methods as described in Materials and methods. Data are means ± SD in three independent experiments. * IGF-I untreated vs. IGF-I treated, p < 0.05; E-cad, E-cadherin; Vim, Vimentin.

To elucidate the effect of IGF-I on human gastric cancer cells, cultured MGC-803 and SGC-7901 cells were treated with recombinant IGF-I (100 ng/mL) for 48 h according to previous study [6]. Figure 1A depicted the dramatic morphological change associated with IGF-I treatment, from characteristic epithelial cells with tight junctions to elongated and spindle-shaped mesenchymal cells. Following IGF-I treatment, immunofluorescence staining detected that the distribution of mesenchymal marker Vimentin was polarized with aggregation at the edges of the cells compared to a network throughout the cell cytoplasm in the control group (Figure 1B). Significant down-regulation of the epithelial marker E-cadherin, and up-regulation of Vimentin and transcription factor ZEB2 were revealed under western blotting analysis (Figure 1C). Additionally, IGF-I treatment enhanced cellular migration, with 78 ± 1.5% treated MGC-803 cells vs. 26 ± 4.0% control cells undergoing migration. Similar findings were made in SGC-7901 cells (p < 0.05; Figure 1D). These data confirm that IGF-I induces EMT in gastric cancer cells.

### IGF-I-induced ZEB2 up-regulation was partially regulated by Akt/ERK pathways

**Figure 2:**
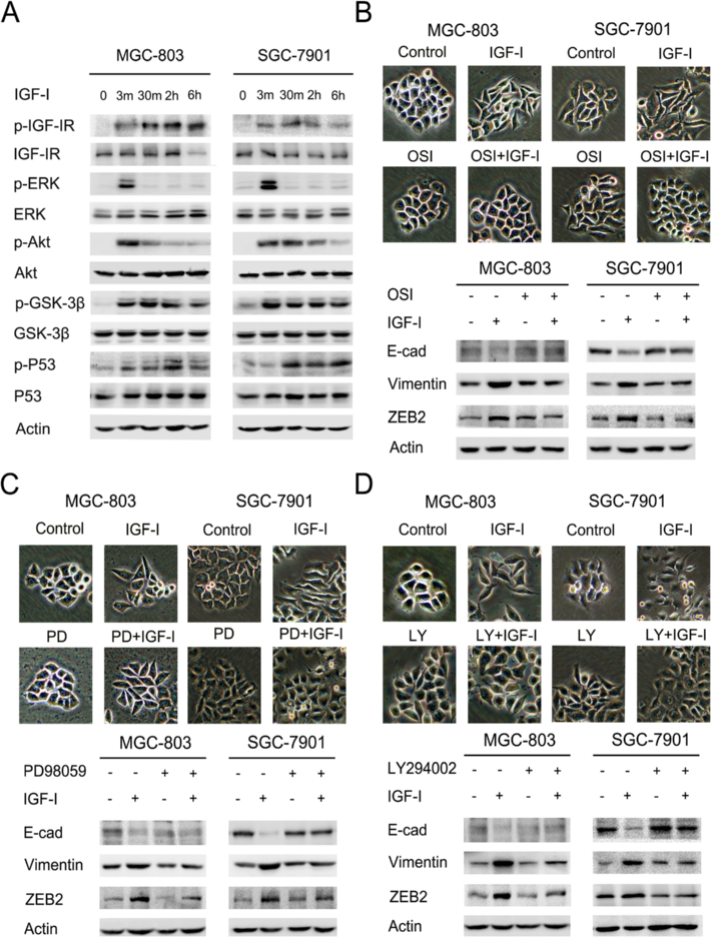
**IGF-I activated Akt/ERK downstream signaling pathways and induced EMT in gastric cancer cells. (A)** The serum-starved MGC-803 and SGC-7901 cells were incubated with IGF-I (100 ng/mL) for the indicated times, the phosphorylation of IGF-IR, ERK and Akt were analyzed by Western blot. **(B-D)** The serum-starved cells were pretreated with or without OSI-906 (10 μM), PD98059 (20 μM) or LY294002 (100 μM) for 2 h followed by IGF-I (100 ng/mL) stimulation for 48 h. Cell lysates were collected for Western blot analysis. Photos were taken at × 20 magnification. E-cad, E-cadherin; OSI, OSI-906; PD, PD98059; LY, LY294002.

Following IGF-I treatment, transient phosphorylation of IGF-IR, Akt, and ERK was detected at 3 min, with recovery to baseline levels after 6 h. Increase in GSK-3β (Ser9) phosphorylation was detected after IGF-I stimulation for 3 min in both MGC-803 and SGC-7901 gastric cancer cell lines. Furthermore, phosphorylation of P53 (Ser15) was detected after IGF-I stimulation for 3 min and 30 min in MGC-803 and SGC-7901 gastric cancer cell lines, respectively (Figure 2A). Cells pretreated with the IGF-IR/IR inhibitor OSI-906 (10 μM) for 2 h before IGF-I stimulation for a further 48 h did not exhibit either morphological changes or epithelial-mesenchymal phenotype marker switching. Additionally, IGF-I-induced ZEB2 up-regulation was prevented in cells pretreated with OSI-906 (Figure 2B). Similarly, the PI3K/Akt inhibitor LY294002 (100 μM) and the ERK inhibitor PD98059 (20 μM) partially suppressed the appearance of IGF-I-induced EMT and ZEB2 up-regulation (Figure 2C, D). Consistently, cell morphology and EMT marker expression represented that IGF-I-induced EMT was also partially reversed after transient knockdown of ERK or Akt gene in SGC-7901 cells (Additional files 1 and 2). These results indicate that IGF-I-induced ZEB2 up-regulation is in part due to activation of the downstream Akt/ERK signaling pathways in gastric cancer cells.

### An Akt/ERK-miR-200c-ZEB2 axis was involved in IGF-I-induced EMT

**Figure 3:**
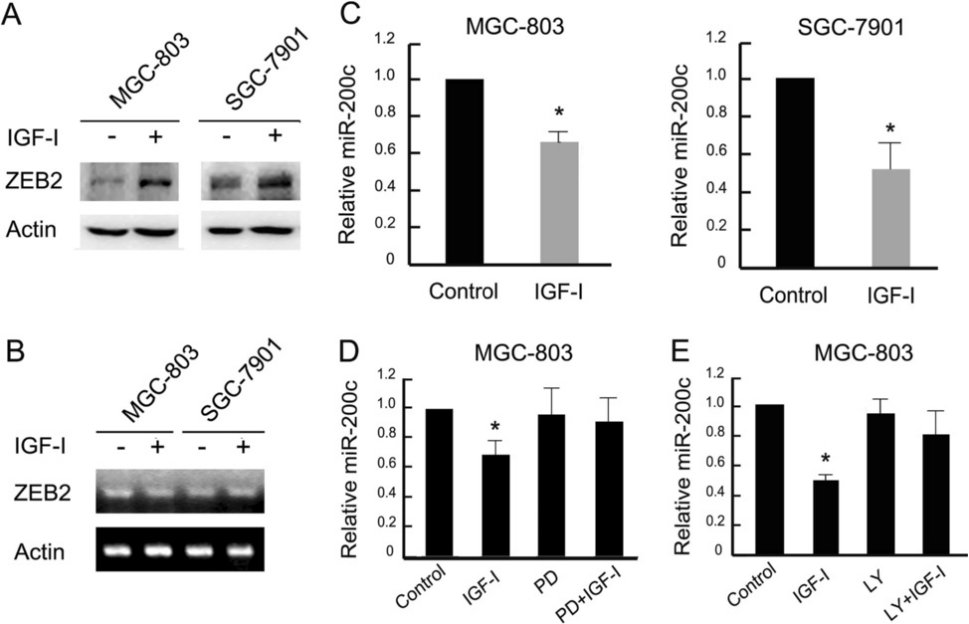
**An Akt/ERK-miR-200c-ZEB2 axis was involved in IGF-induced EMT in gastric cancer cells.** MGC-803 and SGC-7901 cells were serum-starved overnight and then treated with or without 100 ng/mL of IGF-I for 48 h. **(A)** The expression of ZEB2 was analyzed by Western blot. **(B)** The level of mRNA for ZEB2 was analyzed by RT-PCR. **(C-E)** The serum-starved cells were pretreated with or without PD98059 (20 μM) or LY294002 (100 μM) for 2 h followed by IGF-I (100 ng/mL) stimulation for 48 h. The expression of miR-200c was analyzed by real-time PCR. Data are means ± SD in three independent experiments. * IGF-I untreated vs. IGF-I treated, p < 0.05. Control group as reference. PD, PD98059; LY, LY294002.

ZEB2 expression was increased 48 h after IGF-I treatment (Figure 3A). However, quantitative PCR analysis showed no change in ZEB2 mRNA levels with or without IGF-I treatment in both MGC-803 and SGC-7901 cells (Figure 3B). Given that miR-200c represses EMT by targeting ZEB2 [21], we further examined whether the expression of miR-200c was altered in IGF-I-induced EMT. The relative level of miR-200c was decreased by more than 30% in MGC-803 cells and 50% in SGC-7901 cells 48 h following IGF-I treatment (p < 0.05; Figure 3C). Meanwhile, pretreatment with LY294002 and PD98059 partially reversed miR-200c down-regulation after IGF-I stimulation (Figure 3D, E). IGF-I-induced miR-200c decrease was also partially suppressed after transient knockdown of ERK or Akt gene in SGC-7901 cells (Additional file 1C, 2C). These results indicate that an Akt/ERK-miR-200c-ZEB2 axis might be involved in IGF-I-induced EMT in gastric cancer cells.

### Cbl-b repressed IGF-I-induced EMT in gastric cancer cells

**Figure 4:**
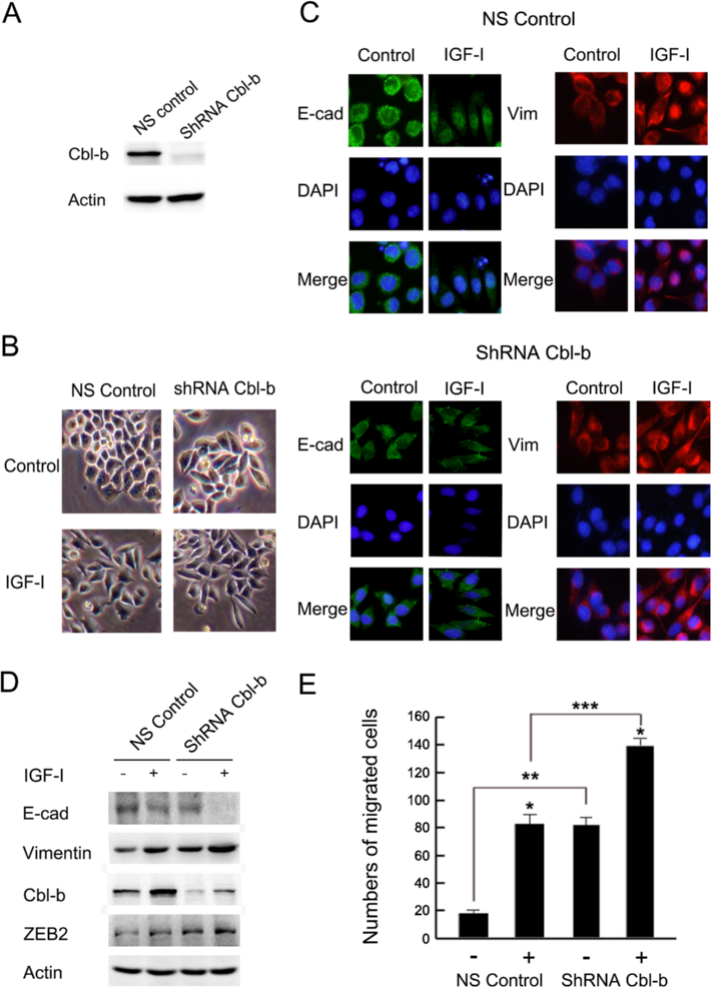
**Cbl-b repressed IGF-I-induced EMT in gastric cancer cells. (A)** Knockdown effect of Cbl-b was examined by Western blot. **(B)** The serum-starved cells were treated with or without IGF-I for 48 h. Photos were taken at × 20 magnification. **(C)** Immunofluorescent staining was performed as described above and visualized by fluorescence microscope at × 40 magnification. **(D)** Cell lysates were collected for Western blot analysis. **(E)** The migration assay was performed using the Boyden chamber methods as described in Materials and methods. Data are means ± SD in three independent experiments. * IGF-I untreated vs. IGF-I treated, p < 0.05; ** In IGF-I untreated group, ShRNA Cbl-b vs. NS Control, p < 0.05; *** In IGF-I treated group, ShRNA Cbl-b vs. NS Control, p < 0.05. E-cad, E-cadherin; Vim, Vimentin; ShRNA Cbl-b, Cbl-b shRNA transfected; NS Control, Non-silencing controls.

Previous study has shown that the Cbl-transforming variant (70z-Cbl) can induce a cascade of molecular alterations leading to EMT [22]. To investigate the effect of Cbl-b on maintaining the epithelial phenotype, shRNA plasmids targeting Cbl-b and non-silencing control plasmids were stably transfected into MGC803 cells. After G418 selection, stable transfectants with Cbl-b expression levels of less than 10% that of endogenous Cbl-b were used in subsequent experiments (Figure 4A). Interestingly, Cbl-b shRNA-transfected (ShRNA Cbl-b) cells lost typical epithelial-like morphology, with acquisition of EMT features. When exposed to IGF-I, ShRNA Cbl-b cells exhibited mesenchymal-like morphology with elongation of the cell shape and cell scattering (Figure 4B). Immunofluorescence detected down-regulation of E-cadherin and up-regulation of Vimentin in Cbl-b knockdown cells when compared with the non-silencing controls (NS Control; Figure 4C). Down-regulation of E-cadherin and up-regulation of Vimentin and ZEB2 were also observed in ShRNA Cbl-b cells in the absence or presence of IGF-I stimulation (Figure 4D). Knockdown of Cbl-b expression enhanced cell migration compared with NS control cells, especially after IGF-I stimulation (Figure 4E). Together, these data suggest that Cbl-b represses IGF-I-induced EMT and maintains the epithelial phenotype in gastric cancer cells.

### Cbl-b maintained the epithelial phenotype through inhibiting the Akt/ERK- miR-200c-ZEB2 axis

**Figure 5:**
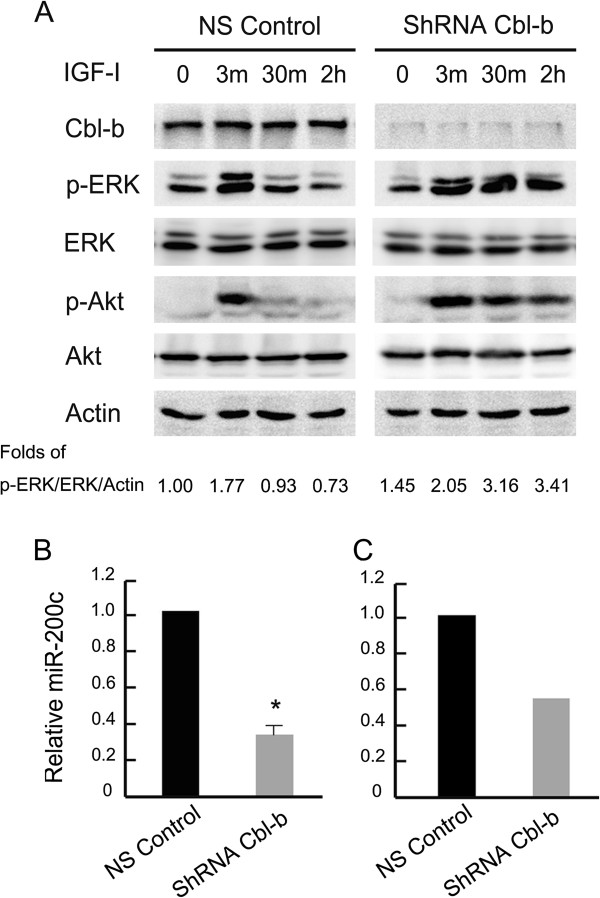
**Cbl-b sustained the epithelial phenotype through inhibiting Akt/ERK-miR- 200c-ZEB2 axis in gastric cancer cell. (A)** The serum-starved cells were treated with or without IGF-I for indicated times, cell lysates were collected for Western blot analysis. **(B)** The expression of miR-200c was analyzed by real-time PCR. **(C)** The expression of miR-200c was performed using microRNA array as described in Materials and methods. Data are means ± SD in three independent experiments. Control group as reference. * ShRNA Cbl-b vs. NS Control, p < 0.05. ShRNA Cbl-b, Cbl-b shRNA transfected; NS Control, Non-silencing controls.

To understand the effect of Cbl-b on Akt/ERK-miR-200c-ZEB2 axis, the activation of Akt/ERK downstream pathways was examined. As shown in Figure 5A, knockdown of Cbl-b prolonged the length of time for Akt and ERK phosphorylation. Additionally, quantitative real-time PCR and microRNA array revealed that the relative level of miR-200c was decreased by 70% and 50% in shRNA Cbl-b cells compared with NS control cells, respectively (Figure 5B, C). These data suggest a role for Cbl-b in sustaining the epithelial phenotype via inhibiting the Akt/ERK-miR-200c-ZEB2 axis in gastric cancer cells.

### Cbl-b inhibited IGF-I-induced EMT by degradation of IGF-IR through the ubiquitin-proteasome pathway

**Figure 6:**
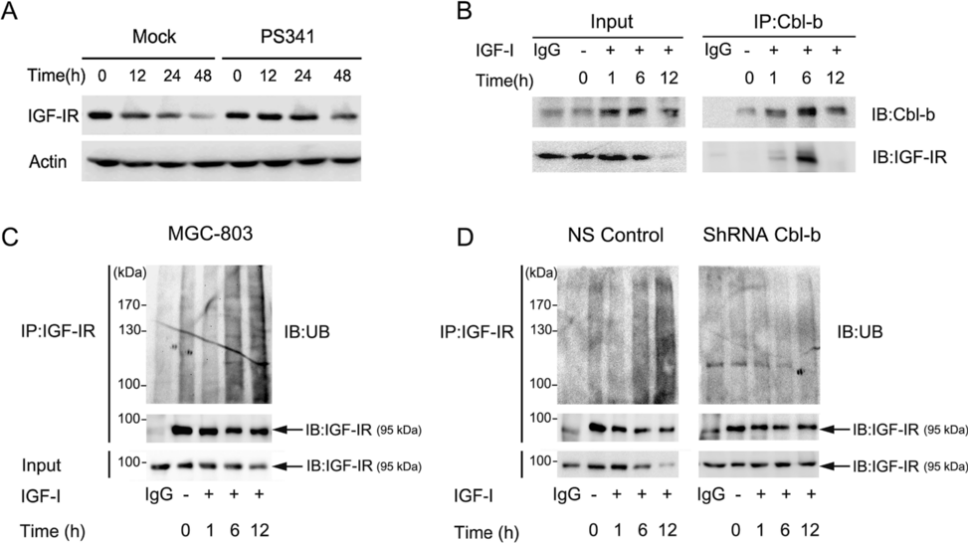
**Ubiquitin ligase Cbl-b ubiquitinated and degradated IGF-IR protein after IGF-I treatment in MGC-803 cell.** The serum-starved MGC-803 cell was pretreated with or without PS341 (5 nM) for 12 hours and then incubated with IGF-I for indicated times. **(A)** The expression of IGF-IR was analyzed by western blot. **(B)** The cell was treated with IGF-I for indicated times before immunoprecipitation with anti-Cbl-b antibody, Cbl-b and IGF-IR were analyzed by western blot. **(C-D)** The serum-starved MGC-803, ShRNA Cbl-b and NS Control cells were treated with IGF-I for indicated times. IGF-IR was immunoprecipitated and ubiquitin was analyzed by western blot. ShRNACbl-b, Cbl-b shRNA transfected; NS Control, Non-silencing controls.

Degradation of IGF-IR protein was observed 48 h after IGF-I treatment in two gastric cancer cell lines (Figure 1C). It has been reported that receptor tyrosine kinases are degraded by receptor ubiquitination, accelerated endocytosis, and lysosome or proteasomal-dependent degradation [23]. To investigate whether the ubiquitin- proteasome pathway mediated the down-regulation of IGF-IR, MGC-803 cells were pretreated with the proteasome inhibitor PS341 (5 nM) for 12 h followed by IGF-I stimulation. IGF-IR degradation was dramatically prevented in these cells (Figure 6A). Given that c-Cbl has an important role in IGF-IR ubiquitination in human osteosarcoma cell lines [24], we next assessed whether Cbl-b could mediate IGF-IR ubiquitination in gastric cancer cells. Cbl-b was associated with IGF-IR 1 h after IGF-I treatment in MGC-803 cells (Figure 6B). A clear increase in ubiquitinated IGF-IR was present 6 h after IGF-I treatment of MGC-803 cells with a peak level reached at 12 h (Figure 6C). This suggested that IGF-I-induced degradation of IGF-IR was dependent on the ubiquitin-proteasome pathway. Furthermore, knockdown of Cbl-b effectively inhibited ubiquitination of IGF-IR compared with the NS control (Figure 6D). Taken together these findings indicate that Cbl-b suppresses IGF-I-induced EMT by ubiquitination and degradation of IGF-IR in gastric cancer cells.

### Association between IGF-IR and Cbl-b expression in gastric cancer tissues

**Figure 7:**
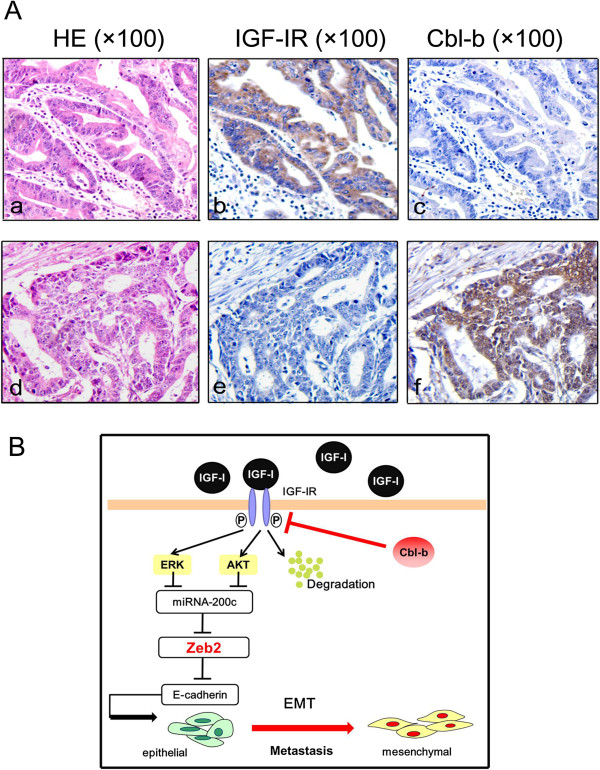
**Representative images for IGF-IR and Cbl-b immunohistochemical staining in three serial sections from two cases. (A) (a, d)** HE staining. The tumor is composed of atypical cells and irregularly shaped tubules. **(b, f)** IGF-IR and Cbl-b positive staining (+) in cell membrane and cytoplasm (in brown). **(c, e)** IGF-IR and Cbl-b negative staining (-). The original magnification is 100×. **(B)** Working model for Cbl-b in IGF-I-induced EMT. In gastric cancer cells, IGF-I binds to its receptor IGF-IR and activates Akt/ERK downstream signaling pathways. The activation of signal pathways up-regulates E-cadherin repressor ZEB2 and down-regulates miR-200c. There is an Akt/ERK-miR-200c-ZEB2 axis in the process. Ubiquitin ligase Cbl-b is required for sustaining the epithelial phenotype probably through targeting IGF-IR for degradation and further inhibiting Akt/ERK-miR-200c-ZEB2 axis in IGF-I-induced EMT.

IGF-IR and Cbl-b expressions were detected using immunohistochemical analysis. Out of 100 gastric adenocarcinoma samples, 69% (69/100) showed IGF-IR positive staining and 58% (58/100) of patients had Cbl-b positive expression. Importantly, there was a significant negative correlation between the expression of IGF-IR and Cbl-b (r = -0.265, p < 0.05) (Table 1). Figure 7A (a-f) showed two representative patients sections of IGF-IR/Cbl-b expression and HE staining. Correlations between the expression of IGF-IR or Cbl-b and the clinicopathological factors were analyzed. As shown in Table 2, more of gastric cancer patients expressing IGF-IR were at late-stage pTNM (p < 0.001) and examined with lymph node metastasis (p < 0.001). On the contrary, patients with Cbl-b expression were more likely to be at early-stage pTNM (p < 0.001) and examined without lymph node metastasis (p < 0.001). However, no associations were found between IGF-IR or Cbl-b expression and age, gender or Lauren grade.

**Table 1:** Spearman’s correlations between Cbl-b expression and IGF-IRexpression in primary gastric cancer patients

**Cbl-b expression**	**Number (%)**	**IGF-IR expression**		**Spearman’s *r*** **P value**	
		**Negative (%)**	**Positive (%)**		
**Negative (%)**	42 (42%)	8 (19.0)	34 (81.0)	-0.265	0.008*
**Positive (%)**	58 (58%)	23 (39.7)	35 (60.3)		
**Number (%)**	100	31 (31%)	69 (69%)		

*P < 0.05.

**Table 2:** Correlation between the expression of IGF-IR or Cbl-b and the clinicopathological factors in primary gastric cancer patients

**Factors**	**All cases**	**IGF-IR**			**Cbl-b**		
		**Negative (%)**	**Positive (%)**	**P value**	**Negative (%)**	**Positive (%)**	**P value**
**Age**^**a**^							
< 59	46	15 (32.6)	31 (67.4)	0.749	22 (47.8)	24 (52.2)	0.278
≥ 60	54	16 (29.6)	38 (70.4)		20 (37.0)	34 (63.0)	
**Gender**							
Male	72	22 (30.6)	50 (69.4)	0.878	31 (43.1)	41 (56.9)	0.733
Female	28	9 (32.1)	19 (67.9)		11 (39.2)	17 (60.8)	
**pTNM stage**							
I + II	46	24 (52.2)	22 (47.8)	<0.001*	6 (13.0)	40 (87.0)	<0.001*
III + IV	54	7 (13.0)	47 (87.0)		36 (66.7)	18 (33.3)	
**Lymph node metastasis**							
Absent	50	26 (52.0)	24 (48.0)	<0.001*	6 (12.0)	44 (88.0)	<0.001*
Present	50	5 (10.0)	45 (40.0)		36 (72.0)	14 (28.0)	
**Lauren grade**							
Intestinal	22	8 (36.4)	14 (63.6)	0.614	7 (31.8)	15 (68.2)	0.452
Diffuse	46	12 (26.1)	34 (73.9)		22 (47.8)	24 (52.2)	
mixed	32	11 (34.4)	21 (65.6)		13 (40.6)	19 (59.4)	

^a^Mean age.

*P <0.05.

## Discussion

The IGF-I/IGF-IR signaling pathway has been reported to induce EMT through activation of the MEK/MAPK and PI3K/Akt pathways in immortalized mammary epithelial, breast, and prostate cancer cells [5,6,25,26]. Furthermore, increased expression of the transcription factor ZEB1 is essential for IGF-I-induced EMT [6]. However, whether IGF-I can induce gastric cancer cell EMT or up-regulate expression of ZEB2, another ZEB transcription factor family member, is unknown. Here, we demonstrated that IGF-I initiated EMT in gastric cancer cells and increased their migration potential through up-regulation of ZEB2. This process was at least partially dependent on Akt/ERK downstream signaling pathways, which were upstream factors of ZEB2 activation in gastric cancer cells in vitro.

ZEB2 is an important transcriptional factor in EMT and functions as a metastasis regulator via direct binding to the promoter site of the cell adhesion molecule E-cadherin in several cancer types [27-29]. Previous studies have identified that an increased ZEB2/E-cadherin ratio positively correlates with invasive disease and poor prognosis in breast and ovarian cancers [30]. Here, we observed that ZEB2 protein levels were up-regulated after IGF-I treatment without an increase in ZEB2 mRNA, suggesting that IGF-I-induced ZEB2 up-regulation might be post-transcriptionally regulated. Recent studies have shown that reduced levels of MicroRNA-200 (miR-200) family members are associated with tumor metastasis and poor disease outcomes [31]. MiR-200c is able to suppress EMT through targeting of ZEB1/2 in some tumor cells [32-34]. Furthermore, the existence of an Akt-miR-200c-E-cadherin axis in the EMT process in renal cell carcinoma has been identified [35]. Here, we found reduced expression levels of miR-200c in both MGC-803 and SGC-7901 gastric cancer cells following IGF-I stimulation. PI3K/Akt inhibitor LY294002, ERK inhibitor PD98059 and transient knockdown of ERK or Akt gene partially reversed the down-regulation of miR-200c by IGF-I. These results support the existence of an Akt/ERK-miR-200c-ZEB2 axis in IGF-induced EMT in gastric cancer cells.

Cbl ubiquitin ligase is reported to maintain AJ dynamics and suppress cell migration through down-regulation of epidermal growth factor receptor-Vav2 signaling in human mammary epithelial cells [16]. Additionally, expression of 70z-Cbl in Madin-Darby canine kidney epithelial cells results in breakdown of cell-cell junctions in a manner characteristic of EMT [22]. Our previous study reported that up-regulation of c-Cbl and Cbl-b was involved in all-trans retinoic acid and bufalin-induced cell adhesion in human promyelocytic cells [17]. This new report revealed that knockdown of Cbl-b facilitated the initiation and progression of EMT in MGC-803 cells. Meanwhile, IGF-induced EMT and migration potential were increased in Cbl-b-knockdown cells. This process was accompanied by prolonged activation time for the Akt/ERK downstream signaling pathways, inhibition of miRNA-200c expression, and up-regulation of the transcriptional repressor ZEB2. Our microRNA array and real-time PCR data reveal a decreased expression level of miRNA-200c in Cbl-b knockdown cells. Together, these findings suggested that Cbl-b repressed IGF-I-induced EMT and migration ability through negative regulation of the Akt/ERK-miR-200c-ZEB2 axis.

To understand how exactly Cbl-b regulated the Akt/ERK-miR-200c-ZEB2 axis in IGF-I-induced EMT, we further investigated the relationship between the Cbl-b and IGF-IR signaling pathways. We found that IGF-IR was first phosphorylated and degraded by IGF-I through the proteasome system. Phosphorylation is the premise of ubiquitination. Phosphorylation of the substrate makes proteins more susceptible to be recognized by the appropriate ligase and formed the ligase complex [36]. Previous study has implicated that EGFR recognition by c-Cbl probably depends on the phosphorylation of a specific receptor tyrosine residue [37]. Consistently, a recent study has reported that c-Cbl combines with IGF-IR and mediates receptor polyubiquitination in response to IGF-I ligand in human osteosarcoma cells [24]. Furthermore, ubiquitination of activated EGFR by c-Cbl complexes is involved in ERβ-1-mediated repression of EMT in basal-like breast cancer cells [38]. In the present study, we found that IGF-IR combined with Cbl-b and initiated IGF-IR ubiquitination after IGF-IR phosphorylation and activation in MGC-803 gastric cancer cells. Combination with Cbl-b initiated receptor degradation of IGF-IR following IGF-I stimulation. Knockdown of Cbl-b significantly inhibited this process. These results suggest that Cbl-b likely ubiquitinates and degrades IGF-IR, and that this is necessary to repress the Akt/ERK-miR-200c-ZEB2 axis and the process of IGF-I-induced EMT. By examining 100 clinical gastric adenocarcinoma tissues, we found that IGF-IR positive expression was significantly associated with late-stage pTNM and positive lymph node metastasis, which was consistent with previous reports [10]. More importantly, the expression of IGF-IR was negatively correlated with the expression of Cbl-b. Cbl-b positive expression was associated with early-stage pTNM and negative lymph node metastasis. These newly reported results further strengthened the possibility that Cbl-b could repress IGF-IR and decrease the risk of developing lymph node metastasis in patients with gastric cancer.

## Conclusions

These findings indicate that IGF-I induces EMT and increases migration ability in gastric cancer cell lines. Furthermore, an Akt/ERK-miR-200c- ZEB2 axis is involved in this process. Cbl-b functions as a crucial repressor in IGF-I-induced EMT through the degradation of IGF-IR and inhibition of the Akt/ERK-miR-200c-ZEB2 axis (Figure 7B). Cbl-b expression is negatively correlated with IGF-IR expression in clinical gastric cancer sample. These results warrant further investigation of the association between the IGF-IR signaling pathway and gastric cancer metastasis. Last, Cbl-b could also serve as a clinical biomarker to identify gastric cancer patients with a lower risk of developing metastasis.

## Abbreviations

EMT: Epithelial-to-mesenchymal transition; IGF-I: Insulin-like growth factor I; IGF-IR: Insulin-like growth factor I receptor; EGFR: Epidermal growth factor receptor; miR-200c: microRNA-200c; AJs: Adherens junctions.

## Competing interests

The authors declare that they have no competing interests.

## Authors’ contributions

YL and XQ designed research; HL and LX performed the data acquisition; CL and LZ supervised the data and algorithms; CL and YM performed data analysis and interpretation; HZ and ZL carried out the statistical analysis; YZ and HL performed immunohistochemistry. RW and ZL performed clinical cases collecting and follow-up. HL performed manuscript preparation; YL and XQ participated in manuscript editing and review. All authors read and approved the final manuscript.

## Supplementary Material

Additional file 1 **Knockdown of ERK reversed IGF-I-induced EMT and decreased level of miRNA-200c. (A-B)** The serum-starved cells were transfected with Scramble Control siRNA or ERK siRNA followed by IGF-I (100 ng/mL) stimulation for 48 h. Cell lysates were collected for Western blot analysis. Photos were taken at × 20 magnification. **(C)** The expression of miR-200c was analyzed by real-time PCR. Data are means ± SD in three independent experiments. * IGF-I untreated vs. IGF-I treated, p < 0.05. Control group as reference. E-cad, E-cadherin. Click here for file

Additional file 2 **Knockdown of Akt reversed IGF-I-induced EMT and decreased level of miRNA-200c.(A-B)** The serum-starved cells were transfected with Scramble Control siRNA or Akt siRNA followed by IGF-I (100 ng/mL) stimulation for 48 h. Cell lysates were collected for Western blot analysis. Photos were taken at × 20 magnification. **(C)** The expression of miR-200c was analyzed by real-time PCR. Data are means ± SD in three independent experiments. * IGF-I untreated vs. IGF-I treated, p < 0.05. Control group as reference. E-cad, E-cadherin. Click here for file
