# Supplementary figures and images for: Ubiquitin ligase Cbl-b represses IGF-I-induced epithelial mesenchymal transition via ZEB2 and microRNA-200c regulation in gastric cancer cells
Source: Mol Cancer. 2014 Jun 2;13:136. doi: 10.1186/1476-4598-13-136 (PMC4052283; doi:10.1186/1476-4598-13-136)

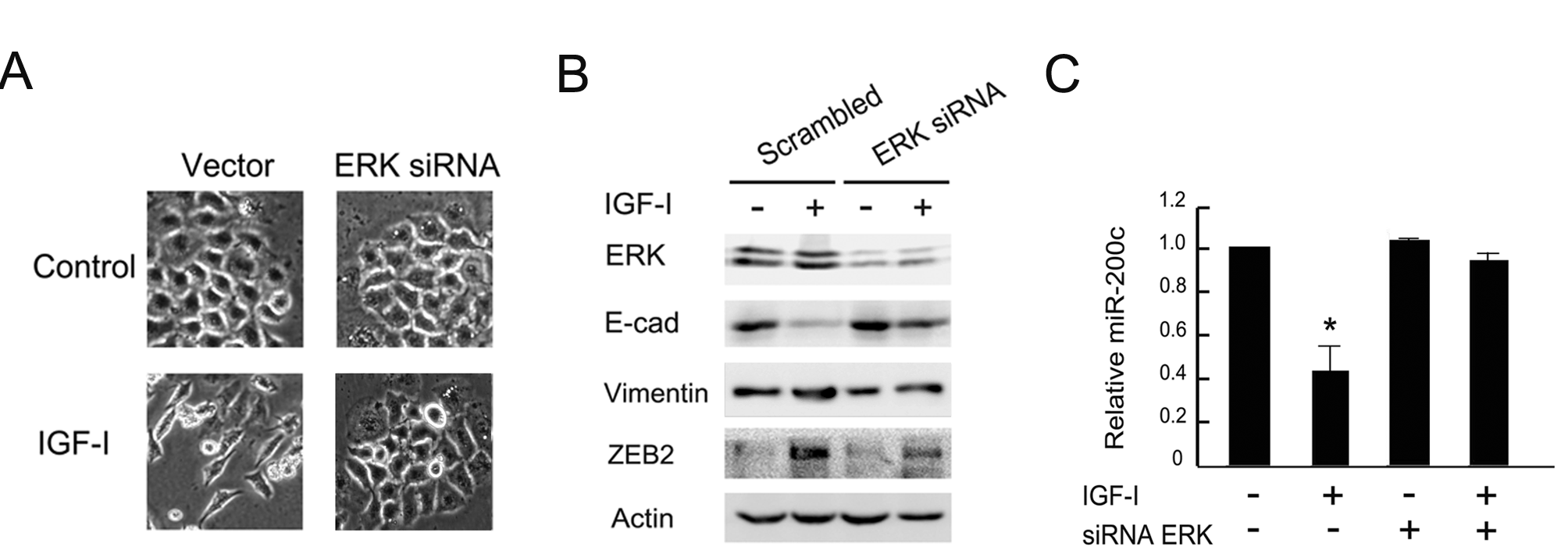

Supplement: Additional file 1 — Knockdown of ERK reversed IGF-I-induced EMT and decreased level of miRNA-200c. (A-B) The serum-starved cells were transfected with Scramble Control siRNA or ERK siRNA followed by IGF-I (100 ng/mL) stimulation for 48 h. Cell lysates were collected for Western blot analysis. Photos were taken at × 20 magnification. (C) The expression of miR-200c was analyzed by real-time PCR. Data are means ± SD in three independent experiments. * IGF-I untreated vs. IGF-I treated, p < 0.05. Control group as reference. E-cad, E-cadherin. [file 1476-4598-13-136-S1.tiff]

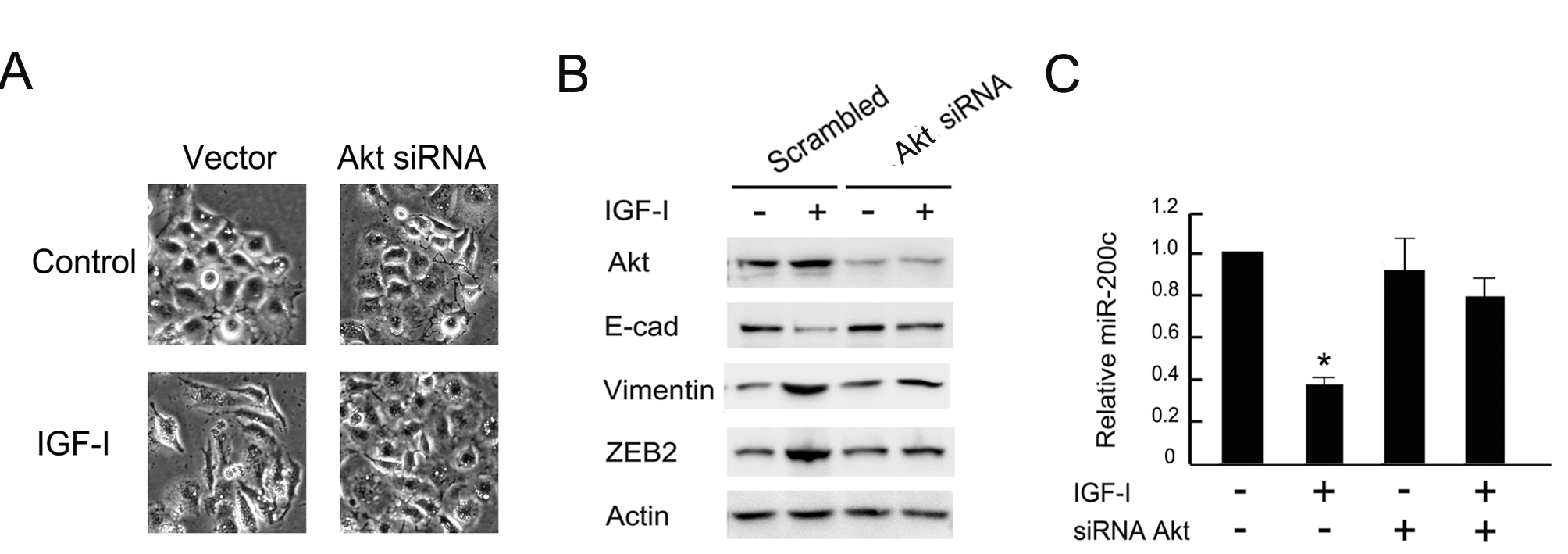

Supplement: Additional file 2 — Knockdown of Akt reversed IGF-I-induced EMT and decreased level of miRNA-200c.(A-B) The serum-starved cells were transfected with Scramble Control siRNA or Akt siRNA followed by IGF-I (100 ng/mL) stimulation for 48 h. Cell lysates were collected for Western blot analysis. Photos were taken at × 20 magnification. (C) The expression of miR-200c was analyzed by real-time PCR. Data are means ± SD in three independent experiments. * IGF-I untreated vs. IGF-I treated, p < 0.05. Control group as reference. E-cad, E-cadherin. [file 1476-4598-13-136-S2.tiff]
